# CT-Angiographic Aspects of Pulmonary Embolism on SARS COV-2

**DOI:** 10.5334/jbsr.3021

**Published:** 2023-04-04

**Authors:** Bénilde Marie-Ange Tiemtore-Kambou, Amadé Ouédraogo, Siaka Ben Aziz Dao, Issouf Franck N’dama Sieba, Adjirata Koama, Idriss Séif Traoré, Salifou Napon, Wilfried Ouédraogo, Harouna Desiré Sankara, Rabiou Cissé, Éric Dienderé

**Affiliations:** 1 Joseph Ki-Zerbo University, BF; 2 Teaching Hospital Bogodogo, BF; 3 Teaching Hospital Bogodogo Ouagadougou, BF; 4 Notre Dame de la paix Polyclinic Ouagadougou, BF; 5 Centre D’imagerie Medicale Ouagadougou, BF

**Keywords:** COVID-19, pulmonary embolism, chest CT angiography, D-dimers

## Abstract

**Objectives:**

To study pulmonary embolism during COVID-19 pneumonia.

**Patients and Methods:**

This was a one-year retrospective and descriptive study of all patients from three imaging sites with SARS-CoV2 infection.

**Results:**

Two hundred and thirty-nine patients were included. The prevalence of pulmonary embolism was 18.4%. The average age was 55 years old. The sex ratio was 1.65. Dyspnea (58.6%), cough (56.1%), and chest pain (40.2%) were the most common reasons for consultation. In 151 patients (63.2%), chest computed tomography (CT) angiography was performed without checking level of D-dimer. The level of D-dimers was elevated in 47.8%. Grade 5 of CO-RADS accounted for 62.3%. In 70.5% of cases, the pulmonary embolism was bilateral with subsegmental involvement in 47.7%.

Condensation in ‘ground glass’ with ‘crazy paving’ were the predominant typical parenchymal lesions with a frequency of 93.7% and 59.4%. In univariate analysis, D-dimers were significantly associated with the occurrence of pulmonary embolism (p < 0.001). Male sex was associated with a non-significantly higher Risk of having a pulmonary embolism (1.18 95% CI: 0.61–2.31, p = 0.622). The critical level increased the risk of pulmonary embolism in a non-significant way. Only the high level of D-dimers was and this, in a significant way.

**Conclusion:**

Pulmonary embolism was increased in the context of SARS-CoV2. The chest CT-angiography associated with the dosage of D-dimers constitutes a good diagnostic arsenal.

## Introduction

The new coronavirus pandemic due to the severe acute respiratory syndrome virus (SARS-CoV-2, also known as COVID-19) emerged in Wuhan, China, in late 2019 and quickly spread around the world.

Chest computed tomography (CT) quickly established itself as the reference imaging in COVID-19 pneumonia [1]. At the early stage, chest CT angiography is not systematic.

Numerous reports have emerged in the literature showing an increased incidence of pulmonary embolism at the segmental and subsegmental levels related to COVID-19 [2].

Chest CT angiography can not only assess the presence of a pulmonary embolism, but also assess the severity of the embolism as well as cardiac function and pressure on the right ventricle. Thus, it could predict the need for admission to the intensive care unit [3].

The aim of this study was to determine the prevalence of pulmonary embolisms on COVID-19 and the correlation of these embolisms with the severity of the disease and the biological marker.

## Patients and Methods

This was a retrospective and descriptive multicenter study from March 9, 2020, to March 31, 2021, carried out in three structural imaging services in the city of xxx. The study population consisted of all patients of all ages, regardless of sex, infected with COVID-19 and presenting for a chest CT angiography. The following patients were included:

- Patients with a positive RT-PCR test for COVID-19 who had performed a chest CT angiography for suspected pulmonary embolism.
- Patients with lung lesions typical of COVID-19 on CT (bilateral and multifocal ground glass opacities predominant in the peripheral, posterior, and basal part), associated with suggestive clinical signs in whom a chest CT angiography was performed for suspicion of pulmonary embolism.

A binary logistic regression analysis was used for the correlation between the presence of pulmonary embolism and our various variables. All examinations were performed using a 16 detector scanner. The CO-RADS (COVID-19 Reporting and Data System) classification was used to assess the degree of suspicion of COVID-19 infection) on CT [4]. The extent of parenchymal abnormalities linked to COVID-19 was carried out according to the degree of parenchymal involvement based on the recommendations of the French Society of Radiology [5].

## Results

**Figure 1:**
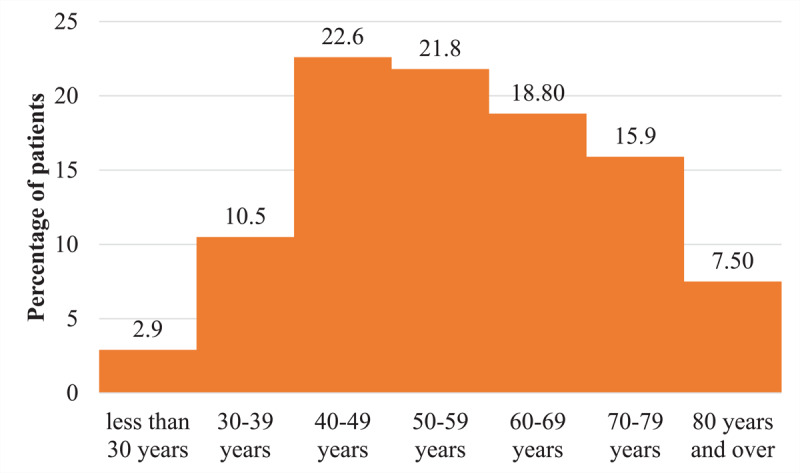
Distribution of patients by age group.

**Figure 2:**
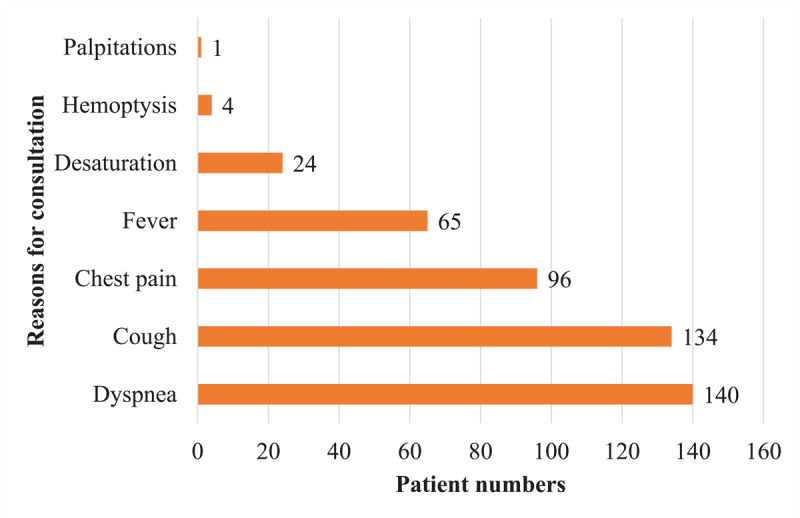
Distribution of patients by reason for consultation.

**Figure 3:**
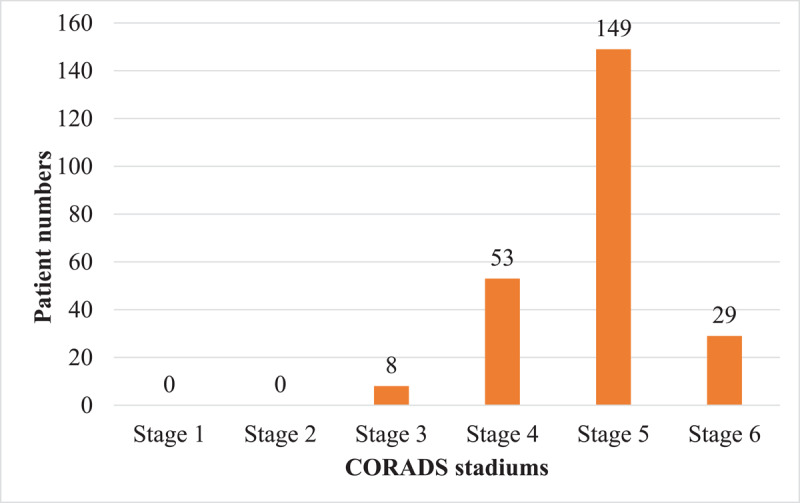
Distribution of patients according to the CO-RADS classification.

**Figure 4:**
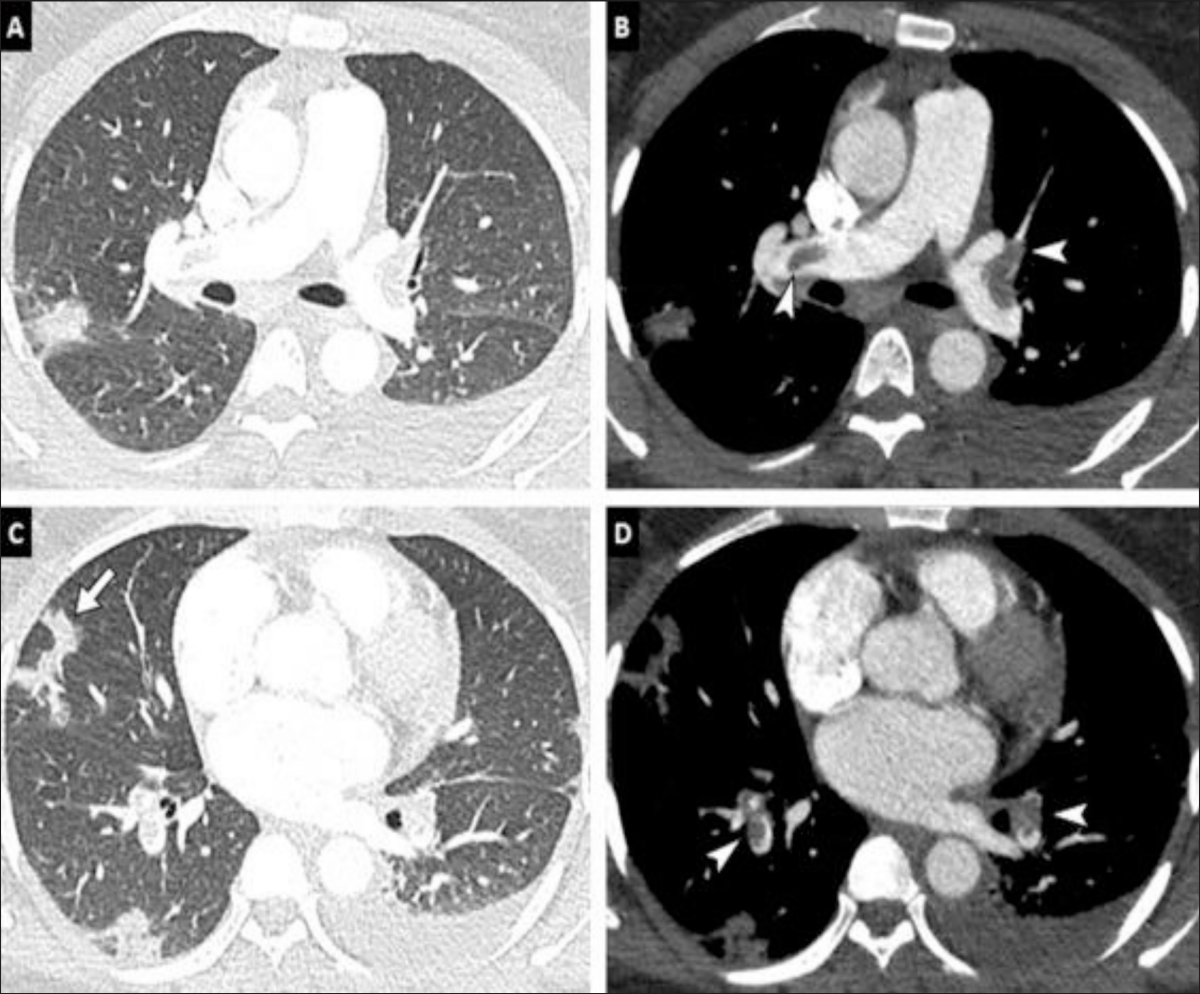
Chest CT-angiography of a 38-year-old patient with no pathological history with a positive PCR for COVID-19 pneumonia. Parenchymal window **(A, C)** shows peripheral foci of condensation. Mediastinal window **(B, D)** finds a bilateral proximal pulmonary embolism (endoluminal defects at the bilateral lobar level) as well as a left pleural effusion.

**Figure 5:**
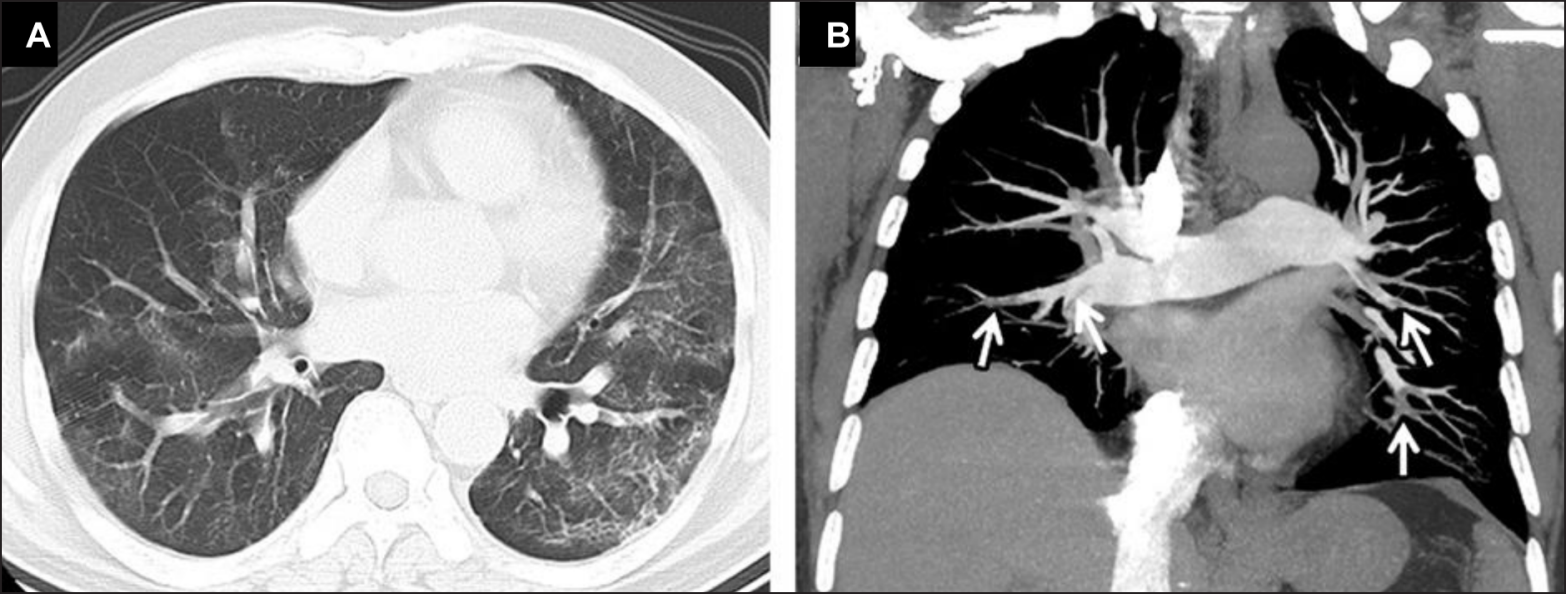
Thoracic CT angiography of a 42-year-old patient with positive PCR for COVID-19. Parenchymal window in the axial section **(A)** without injection shows peripheral bilateral ‘ground glass’ areas (arrow). The mediastinal window **(B)** in coronal reconstruction (MIP), reveals several bilateral endoluminal defects involving the lobar, segmental, and sub-segmental branches of the pulmonary artery (arrow).

During the period of our study, 260 chest CT angiographies for suspicion of pulmonary embolism were performed, of which 239 (91.92%) met our eligibility criteria. The prevalence of pulmonary embolism was 18.4% (44/239) [95% CI: 13.7%–23.9%]. Patients came from the emergency department in 47.3% (113/239) (Table 1). The average age was 55 years old with extremes of 17 and 96 years old. The age group ranging from 60–80 years old represented 42.2% (101/239), shown in Figure 1. Men represented 62.3% (149/239), with a sex ratio (M/F) of 1.65. Dyspnea (58.6% [140/239]), cough (56.1% [134/239]), and chest pain (40.2% [96/239]) were the most common reasons for consultation. Figure 2 shows these different patterns. Among patients, 2.3% (5/239) had at least one risk factor. Arterial hypertension, bronchopneumopathies, renal insufficiency, and diabetes were the most common comorbidities, with a cumulative frequency of 17.1%. In 151 (63.2%) patients, chest CT angiography was performed without checking the level of D-dimer. The level of D-dimers was high in 47.8% (114/239) of patients who had levels checked. All patients with embolism had an elevated level of D-dimers. All the collected cases had a CT scan meeting our required quality criteria. CO-RADS 5 was the most represented with 62.3% (149/239) (Figure 3). In 70.5% (31/44) of the cases, the pulmonary embolism was bilateral (Figure 4), and the embolism was located at the sub-segmental level in 47.7% (21/44) of the cases (Figure 5). The degrees of damage from severe (21.3%) to critical (10.5%) represented 31.8% (76/239) of all the parenchymal lesions of the French Society of Radiology classification. The other grades were divided into: minimal (20.5% [49/239]); moderate (21.8% [52/239]); and extensive (25.9% [62/239]). Condensation in ‘ground glass’ with ‘crazy paving’ were the predominant parenchymal lesions with a frequency of 93.7% (224/239) and 59.4% (142/239) (Table 2). There were 28 patients with signs of severity associated with pulmonary embolism (63.6%). These patients had a dilation of the right heart chambers in 32.1% (9/28) of cases; PAH in 37.7% (10/28) of cases; pericardial effusion in 25% (7/28) of cases; and pleural effusion in 2 cases. In univariate analysis, D-dimers were significantly associated with the occurrence of pulmonary embolism (p < 0.001). Thus, patients with a positive D-dimer test were about 50 times more likely to develop a pulmonary embolism than those with a negative test. Male sex was associated with a non-significantly higher Risk of having a pulmonary embolism (1.18 95% CI: 0.61–2.31, p = 0.622). Moderate, extensive, and severe levels of lung damage were not associated with the occurrence of embolism, while the critical level of lung damage increased this risk; however, this relationship was not statistically significant. In general, history of chronic obstructive pulmonary disease (0.48 95% CI: 0.06–3.90, p = 0.493), arterial hypertension (0.57 95% CI: 0.13–2.59, p = 0.469), and renal failure (0.88 95% CI: 0.10–7.76, p = 0.911) were not associated with the occurrence of pulmonary embolism. D-dimers were only performed in 88 patients (36.82%), so the variable could not be included in the fitted model. In multivariate analysis, no variable was statistically associated with the occurrence of pulmonary embolism. These different associations are represented by Table 3.

**Table 1:** Distribution of cases by department of origin.


ORIGIN	WORKFORCE	PERCENTAGE

**Emergency**	113	47.3

**Intensive care unit**	59	24.7

**Others**	37	15.5

**Not specified**	30	12.5

**Total**	**239**	**100**


**Table 2:** Distribution according to the type of parenchymal lesion.


LESIONS	WORKFORCE	FREQUENCY (%)

Frosted glass	224	93.7

Crazy paving	142	59.4

Reverse halo	41	17.2

Traction bronchiectasis	35	15.5

Vascular dilation to the crazy paving	27	11.3

Parenchymal condensations	18	7.7


**Table 3:** Factors associated with the presence of a pulmonary embolism.


VARIABLES	UNIVARIATE BINARY LOGISTIC REGRESSION		MULTIVARIATE BINARY LOGISTIC REGRESSION	
	
	OR [95% CI]	P-VALUE	AOR [95% CI]	P-VALUE

**Sex**				

Male	1		1	

Feminine	1.18 [0.61–2.31]	0.622	1.15 [0.58–2.27]	0.696

**Age**	1.02 [0.99–1.04]	0.158	1.02 [0.99–1.04]	0.149

**History of COPD**				

No	1		1	

Yes	0.48 [0.06–3.90]	0.493	0.46 [0.05–3.90]	0.477

**History of hypertension**				

No	1		1	

Yes	0.57 [0.13–2.59]	0.469	0.55 [0.12–2.55]	0.443

**RI history**				

No	1		1	

Yes	0.88 [0.10–7.76]	0.911	0.81 [0.09–7.47]	0.851

**Severity of parenchymal lesions**				

Absent/minimal	1			

Moderate	0.45 [0.15–1.33]	0.149	0.46 [0.15–1.39]	0.170

Extent	0.75 [0.29–1.90]	0.538	0.72 [0.28–1.87]	0.496

Strict	0.74 [0.28–1.98]	0.549	0.68 [0.25–1.86]	0.453

Critical	1.34 [0.45–4.04]	0.599	1.25 [0.41–3.84]	0.696

**D-dimers**				

Negative	1		–	–

Positive	49.50 [6.23–393.09]	<0.001*	–	–


## Discussion

The prevalence of pulmonary embolism in our series was 18.4%. This confirms the increased nature of pulmonary embolism in SARS-CoV2. This result is similar to that of Coulibaly, who described 18.2% of cases in patients with COVID-19 coming exclusively from intensive care units [6]. The high prevalence would be explained, according to several studies, by a systemic endothelite with a severe attack of the microcirculation, responsible for a vasoconstriction and micro-thromboses [7,8]. Our result remains, however, lower than that of certain authors of the literature, in particular, Western literature. Indeed, and Ventura-Díaz found a higher prevalence than ours, of 26% and 33%, respectively [9,10]. The male predominance was also found by Grillet [11] and Parrek [12] in, respectively, 70% and 52.6% of cases. This result could be explained by the fact that men are more affected by COVID-19 infection in the general population and die from it more [13]. Table 4 allows a comparison of the presence of comorbidities (hypertension and diabetes) according to the authors [6,11,10,14,15,12]. In our study, the reason for consultation was dominated by dyspnea (58.6%), cough (56.1%), and chest pain (40.2%). The reasons for consultation in our study were similar to those of Hoda [16] and Chen [17]. D-dimer levels were elevated in all cases with pulmonary embolism, similar to the results seen by Hoda [16], Leonard-Lorant [18], Chen [17], and Helms [19], who associated higher D-dimer levels in all cases of COVID-19 field pulmonary embolism. A natural increase in D-dimer is observed during COVID-19, with a higher tendency in cases of pulmonary embolism [17,6]. Also, our data did not allow us to define a precise threshold at risk of occurrence of a pulmonary embolism; nevertheless, the level of D-dimers constitutes a parameter that can help the practitioner to properly prescribe CT angiography, or even anticoagulant prophylaxis as recommended by certain studies [12,19].

**Table 4:** Comparison of the presence of comorbidities according to the authors in percentage.


AUTHORS	OUR STUDY	COULIBALY [6]	GRILLET [11]	LODIGIANI [10]	CUI [14]	SILVA [15]	PAREEK [12]

**Hypertension**	**7.1**	31.8	39	47.2	48	58	60.2

**Diabetes**	**2.1**	19	20	22.7	25	23.9	39.6


The subsegmental seat of the emboli was comparable to those described by Leonard-Lorant [18], Chen [17], and Robert [20], who detected peripheral localization of pulmonary embolism with a respective frequency of 44%, 70%, and 65.3%.

The high frequency of peripheral pulmonary embolism suggests that local thrombosis may play a more important role in the development of pulmonary embolism in COVID-19, rather than classic thromboembolism in patients without COVID-19 [8]. This hypothesis is supported by an in vivo chest CT study highlighting an endothelitis with local micro-thromboses [21]. Concerning the degree of severity of parenchymal involvement, our data differ from those of Freund [22] (international multicentre study) and from Bompard [2], who found 35% and 51% of severe lesions respectively. Would this be due to the low number of serious cases received in hospitals in our context?

In univariate analysis, the high level of D-dimer was significantly associated with the occurrence of pulmonary embolism (p < 0.001), confirmed by several publications [17,14,23]. Age and classic comorbidities were not associated with the occurrence of pulmonary embolism. This is similar to the results of Chen, Cui, and Fauvel [17,14,24], suggestting a particular pathophysiological mechanism of pulmonary embolism in the context of SARS-CoV2. Additionally, biomarkers of inflammation, heart and muscle damage, liver and kidney dysfunction, and clotting measurements were also found to be significantly elevated in patients with both severe and fatal COVID-19 [25]. In multivariate analysis, no variable was statistically associated with the occurrence of pulmonary embolism in our study. Other large-scale investigations are needed to confirm the determinants of this pathology in our context.

## Conclusion

The prevalence of pulmonary embolism in SARS-CoV2 is increased, to 18.4%, and generally concerns patients admitted to intensive care. Only the high level of D-dimer was significantly associated with this thromboembolic phenomenon. The dosage of D-dimers is therefore a good parameter to help prescribe CT angiography and possible anticoagulant prophylaxis.

A large-scale study is necessary to define a precise threshold for the level of D-dimers at risk of occurrence of a pulmonary embolism.
