# Correction: CT-Angiographic Aspects of Pulmonary Embolism on SARS COV-2

**DOI:** 10.5334/jbsr.3202

**Published:** 2023-05-12

**Authors:** Benilde Marie-Ange Tiemtoré-Kambou, Amadé Ouédraogo, Siaka Ben Aziz Dao, Issouf Franck N’dama Sieba, Adjirata Koama, Idriss Séif Traore, Salifou Napon, Éric Arnaud Diendéré, Wilfried Ouédraogo, Harouna Desire Sankara, Rabiou Cisse

**Affiliations:** 1 Department of Medical Imaging and Interventional Radiology, Teaching Hospital Bogodo, Ouagadougou, Burkina Faso; 2 UFR SDS, University Joseph Ki-Zerbo, Ouagadougou, Burkina Faso; 3 Department of Radiology Teaching Hospital, Yalgado Ouedraogo, Ouagadougou, Burkina Faso; 4 Intensive Care Unit, Teaching Hospital of Bogodo, Ouagadougou, Burkina Faso; 5 Emergency Department Teaching Hospital Bogodo, Ouagadougou, Burkina Faso; 6 Infectious Disease Department, Teaching Hospital Bogodo, Ouagadougou, Burkina Faso; 7 Notre Dame de la paix Polyclinic, Ouagadougou, Burkina Faso; 8 Medical Imaging Centre of Ouagadougou, Burkina Faso

**Keywords:** COVID-19, pulmonary embolism, chest CT angiography, D-dimers

## Abstract

This article details a correction to the article: Tiemtore-Kambou BM-A, Ouédraogo A, Dao SBA, Sieba IFN, Koama A, Traoré IS, Napon S, Ouédraogo W, Sankara HD, Cissé R, Dienderé É. (2023). CT-Angiographic Aspects of Pulmonary Embolism on SARS COV-2. *Journal of the Belgian Society of Radiology*, 107(1): 22, 1–8. DOI: https://doi.org/10.5334/jbsr.3021.

## Correction

The authors of the article ‘CT-Angiographic Aspects of Pulmonary Embolism on SARS COV-2’ have decided to make an amendment to the order of authorship for this article, listing Professor Rabiou Cissé last as he is the lead author for the article, and also due to his seniority. The updated reference for the article will be Tiemtore-Kambou BM-A, Ouédraogo A, Dao SBA, Sieba IFN, Koama A, Traoré IS, Napon S, Ouédraogo W, Sankara HD, Dienderé É, Cissé R. (2023) [1]. CT-Angiographic Aspects of Pulmonary Embolism on SARS COV-2. *Journal of the Belgian Society of Radiology*, 107(1): 22, 1–8. DOI: https://doi.org/10.5334/jbsr.3021.
